# Prevalence of Underweight, Overweight, and Obesity in Adults in Bhaktapur, Nepal in 2015–2017

**DOI:** 10.3389/fnut.2020.567164

**Published:** 2020-09-22

**Authors:** Catherine Schwinger, Ram K. Chandyo, Manjeswori Ulak, Mari Hysing, Merina Shrestha, Suman Ranjitkar, Tor A. Strand

**Affiliations:** ^1^Department of Global Public Health and Primary Care, Centre for Intervention Science in Maternal and Child Health, Centre for International Health, University of Bergen, Bergen, Norway; ^2^Department of Community Medicine, Kathmandu Medical College, Kathmandu, Nepal; ^3^Department of Psychosocial Science, Faculty of Psychology, University of Bergen, Bergen, Norway; ^4^Department of Research, Innlandet Hospital Trust, Lillehammer, Norway

**Keywords:** nutritional status, anthropometry, double burden of malnutrition, low income country, Asia

## Abstract

**Introduction:** There is an increase in the double burden of malnutrition globally, with a particular rise documented in Asia. In Nepal, undernutrition has been prevalent for decades. Today, however, the incidence of overweight and obesity (OWOB) in the country has increased substantially. There is a need to conduct local studies reporting on the concurrent burden of both underweight and OWOB across adult populations. This study addresses this need by describing the distribution of body mass index (BMI) in a defined population of adults living in the peri-urban community of Bhaktapur, Nepal.

**Material and methods:** For this cross-sectional analysis, we used data that were available from 600 women and 445 men whose children were enrolled in an individually randomized, double-blind, placebo-controlled trial assessing the effect of daily vitamin B12 supplementation. Upon enrolment of their 6–11-month old children, mothers and fathers were interviewed about their socio-demographic details. In addition, their weight and height were measured by trained field workers. Each parent's BMI was calculated as the ratio of body weight (in kg) and height squared (in m), expressed as kg/m^2^, and categorized according to the WHO recommendation. We used linear and multinomial logistic regression models to assess associations between the BMI of the mothers and fathers, and their baseline characteristics.

**Results:** The mean BMI was 23.7 kg/m^2^ for both the mothers and fathers with a standard deviation (SD) of 3.6 and 3.7, respectively. The proportion categorized as underweight, overweight, and obese was also similar in the two groups with around 5% being underweight, 30% being overweight and 5% being obese. Age was positively associated with BMI in both groups. Those categorized as daily wage earner had a lower mean BMI than those in other occupational groups.

**Conclusion:** Our results contribute to documenting the burden of both under- and overnutrition in a selected group of young adults living in a peri-urban community in Nepal. As Nepal is undergoing an improvement in its economic situation, as well as a nutrition transition, it is important to provide sufficient information to enable timely action, and evidence-based decision-making to prevent a further increase in Nepal's growing double burden of malnutrition.

## Introduction

Globally, in 2015, it was estimated that 9% of the world's adult population were underweight and 30–40% were overweight or obese (1, 2). Women had a slightly higher prevalence of overweight and obesity (OWOB) (1). Although there has been a marginal decline in the prevalence of underweight (1), the rise in the proportion of people being overweight or obese is currently being described as a global pandemic. There has been a 50–80% increase in OWOB in the past 30 years (2). Despite some variability, the increase in the prevalence of OWOB is seen across all countries, ages, socio-economic strata, and in both sexes.

Both underweight and overweight affect the functioning as well as structure of body organs, increasing the risk of mortality (3, 4). A nutritional status outside of the normal range is one of the leading risk factors for premature death and loss of disability adjusted life years (DALY) (5). Overweight and obesity are associated with several other adverse health outcomes, including diabetes mellitus type 2, cardiovascular disease, certain types of cancer, musculoskeletal and mental disorders as well as pregnancy complications (3, 6, 7). Amongst others, undernutrition is associated with an increased risk of infection (8) as well as pre-term birth/ low birth weight (7, 9).

Malnutrition, in all its different forms, affects all countries of the world (10). Many countries face a double burden of malnutrition (DBM), where both undernutrition and OWOB exist in the same population, household, or even individual (11). While in high-income countries, OWOB is usually more prevalent among populations with a low socio-economic status (SES), the opposite has been observed for low-income countries (2, 12, 13). The results from a recent analysis including 126 low- and middle-income countries (LMIC) showed that the increase in the global DBM was driven by the countries with a low gross domestic product, as these tended to have a greater increase in OWOB and a slow decrease in the prevalence of undernutrition. This increase in the DBM was particularly observed in Asia (11).

Studies undertaken in Nepal have shown that although the prevalence of undernutrition in the population has generally decreased over the last decades, it has remained high among women of reproductive age. During this period the prevalence of OWOB has increased substantially (14, 15). Three national surveys (Demographic Health Survey (DHS) Nepal 2016, Micronutrient Survey 2016 and the STEPS survey 2019), estimated that overall 14.5–17% of the Nepalese adult women were underweight, while 22–25% were OWOB (16–18). Among men, 17% were underweight and 17–23.4% were OWOB (16, 17). The increase in OWOB is an important driver for the increase in the DBM in Nepal (11). In the Nepal DHS 2016, there was no difference in the prevalence of OWOB between ecological zones or between rural vs. urban populations (19). However, Nepal is a country with a large diversity in both populations and ecology, and thus nation-wide estimates or even broad comparison groups such as ecological zones will dilute important local differences. It is important to remember that the 2020 Lancet series on the DBM stresses the importance of describing the full distribution of anthropometric status of populations instead of assessing undernutrition and OWOB in their own silos (11, 20–22). Currently, there are a few local studies on the prevalence of OWOB in Nepal (23–25); however, these do not report on the burden of undernutrition in the same population.

We used high quality data on the anthropometric status from young adults whose child was enrolled in a community-based trial. Participants lived in the peri-urban municipality of Bhaktapur, Nepal. The study period was from 2015–2017. Our aim was to describe the whole distribution of BMI and the prevalence of both underweight, overweight, and obesity in this poor population, including associated factors.

## Materials and Methods

### Study Setting

The study was conducted in the municipality of Bhaktapur, located 15 km east of Kathmandu, Nepal's capital. According to the 2011 census, the municipality had a total population of around 82,000 (26). It is 1,300 m above sea level and its sub-tropical climate is characterized by a warm and wet monsoon season (June–September) and a cold and dry winter season (December–February). The main livelihood is agriculture. Other important sources of income are small-scale self-owned businesses and daily wage labor. The diet of people in this municipality tends to be simple. They consume large amounts of rice, which may help to explain why their diet is generally low in adequate levels of micronutrients (27).

### Study Design and Data Collection

For this cross-sectional analysis, we used data from a community-based, randomized, double-blind, placebo-controlled trial. The aim of the trial was to assess the efficacy of supplementing infants daily with 2 μg vitamin B12 over a period of 12 months on neurodevelopment, growth and hemoglobin concentration. Enrollment took place between April 2015 and February 2017. A total of 600 infants were included, aged 6–11 months with a length-for-age <-1 *Z*-score. Other inclusion criteria included plans to reside in the study area for the next 12 months, and an available informed consent from the caregiver(s). Details about exclusion criteria, randomization and intervention can be found in the trial protocol (28).

Upon enrolment, both mothers and fathers of the participating child were interviewed about their socio-demographic details. These included age, educational attainment level, occupation, smoking, alcohol consumption, number of pregnancies and family members, family type, and caste. Educational level was divided into illiterate, primary school, secondary school, SLC/intermediate school, bachelor's degree, and above. For occupation, we used the following groups: daily wage earner, no work/agriculture, carpet worker, self-employed, working in the services industry, and working abroad. The categories no work and agriculture were combined as persons in this group did not have a formal employment despite working on their own fields. Fathers were asked if they smoked; for mothers, we used the general variable for indoor smoking (yes/no). Only fathers were asked about their alcohol consumption (yes/no). Mothers were asked how many pregnancies they have had (gravida). For this analysis, we categorized the number into 1 and ≥2. Family type was either nuclear or joint, where nuclear was defined as only the parents and their children living together. Caste was categorized into Newar, Bramhin, Chhetri, Tamang/Lama, and others. The parents' weight and height were measured by trained field workers at a clinic using a portable electronic scale (Salter/HoMedics Group, UK and seca, Germany) with a precision of 100 g and a stadiometer (Prestige, Hardik Medi Tech, India) with a precision of 1 mm, respectively. The scales and the stadiometer were calibrated regularly. The parents were asked to remove their shoes and any heavy clothing before the anthropometric measurements.

### Definition Outcome

As indicator of the parents' nutritional status, we calculated body mass index (BMI) as the ratio of body weight (in kg) and height squared (in m), expressed as kg/m^2^. We categorized their nutritional status according to the WHO recommendation for underweight (BMI <18.5), normal weight (18.5–24.9), overweight (BMI 25–29.9) and obesity (BMI ≥30). For Asian populations, a WHO expert panel has suggested additional trigger points (in addition to the commonly used cut-off points). These include a BMI ≥23 kg/m^2^ as representing increased risk, and a BMI ≥27.5 kg/m^2^ as representing high risk. These trigger points were based on different associations between BMI and risk for cardiovascular disease and diabetes mellitus type 2 as observed in Asian populations (29).

### Statistical Analysis

All analyses were done in Stata (version 15). Characteristics of the participants included in the analyses are described as mean (SD), median (IQR) or proportion as appropriate.

The BMI of mothers and fathers is reported separately as mean (SD). The proportions are categorized as underweight, overweight, and obese. We created density plots to depict the distribution of BMI among mothers and fathers separately. To assess the association between the BMI of the mothers and fathers with their baseline characteristics, we used linear regression models with BMI as continuous outcomes. Those variables found to be statistically significant at a level of 0.2 were entered simultaneously into a multivariable model. Variables were retained in the final model if they were found to be statistically significant at a level of 0.05. The same procedure was done for the multinomial logistic regression models. In these models, we used the normal BMI range as a reference and the categories of undernutrition as well as OWOB as comparison groups. Results from these models are reported as relative risk (RR).

To compare the characteristics of those fathers with and without data on BMI, we used a two-sided *t*-test for the continuous variables and a Chi^2^ Test for the dichotomous variables.

### Ethics

Ethical clearance for the trial was obtained from the Nepal Health Research Council (NHRC #233/2014) and from the Regional Committee for Medical and Health Research Ethics (REC # 2014/1528) in Norway. The parents were thoroughly informed about the study, and their written informed consent was obtained. In the case of illiteracy, a thumbprint was given by the caretaker in the presence of an impartial witness to signify informed consent. All activities conformed to the Declaration of Helsinki.

## Results

In total, weight and height were measured for 600 mothers and 445 fathers. In general, the fathers were slightly older (mean 30.2 vs. 27.3 years). The majority of the were unemployed (58%), while only 2.7% of the fathers were without work. Of the 322 fathers who reported drinking alcohol, 70 (22%) reported that this involved drinking daily, and 139 (43%) reported drinking once a month or less. Most of the participants were from the Newar caste (70%). Details of the participants' characteristics are shown in Table 1.

**Table 1:** Characteristics of study participants included in the analysis.

**Characteristic**	**Mothers (*n* = 600)**	**Fathers (*n* = 445)**
Mean age, years (SD)	27.3 (4.6);	30.3 (5.2);
	range 17–43	range 18–54
**Education level**, ***n*** **(%)**		
Illiterate	48 (8.00)	10 (1.7)
Primary	175 (29.2)	202 (33.7)
Secondary	113 (18.8)	131 (21.9)
SLC/Intermediate	148 (24.7)	149 (24.9)
Bachelor's degree	90 (15.0)	74 (12.3)
Above	26 (4.3)	33 (5.5)
**Occupation**, ***n*** **(%)**		
No work/ agriculture	373 (62.3)	28 (6.3)
Carpet worker	17 (2.8)	6 (1.4)
Daily wage earner	73 (12.2)	166 (37.3)
Self employed	75 (12.5)	137 (30.8)
Services	61 (10.2)	94 (21.1)
Working abroad	0	14 (3.1)
Smoking (yes), *n* (%)	303 (50.5)	247 (55.5)
Alcohol consumption (yes), *n* (%)		322 (72.4)
**Gravida**, ***n*** **(%)**		
Primi	280 (47)	
≥2	320 (53)	
**Family type**, ***n*** **(%)**		
Nuclear	308 (51.3)	222 (49.9)
Joint	292 (48.7)	223 (50.1)
Median number of family	5 (4–7); range: 2–13	5 (4–7); range: 3–13
members (IQR)		
**Caste**, ***n*** **(%)**		
Newar	422 (70.3)	336 (75.5)
Brahmin	20 (3.3)	11 (2.5)
Chhetri	27 (4.5)	17 (3.8)
Tamang/Lama	91 (15.2)	54 (12.1)
Others	40 (6.7)	27 (6.1)

**Figure 1:**
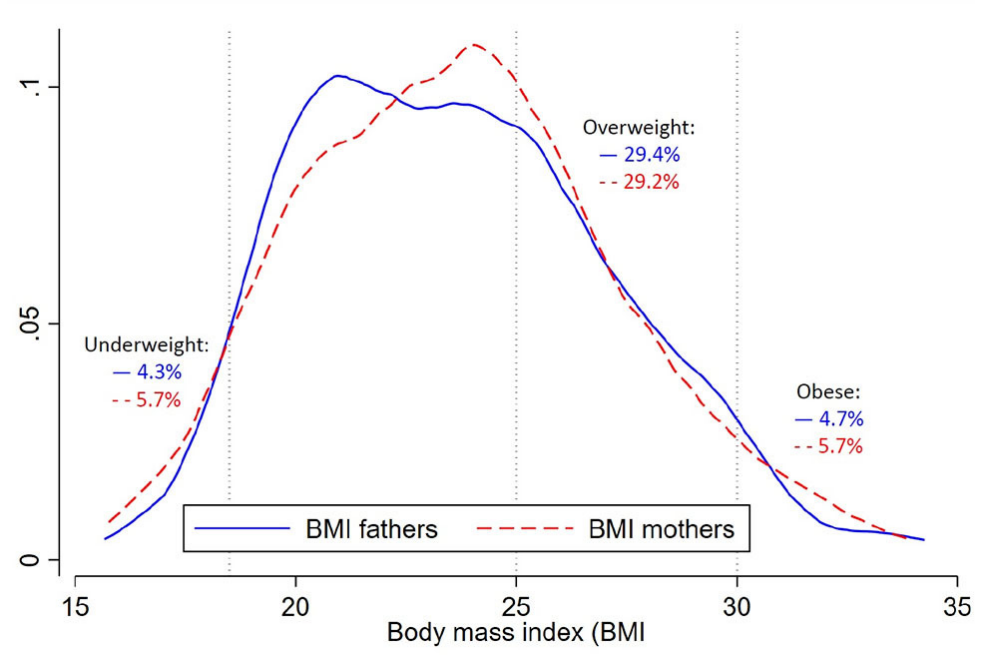
Density plot for the body mass index (BMI; in kg/m^2^) and prevalence of underweight, overweight and obesity of fathers (solid blue line, *n* = 445) and mothers (dotted red line, *n* = 600). Underweight is defined as BMI <18.5, overweight as BMI 25–29.9 and obesity as BMI ≥30.

The mean BMI of the mothers and fathers were similar, 23.7 kg/m^2^ for both (SD 3.6 and 3.7, respectively). The distributions with the commonly used WHO cut-offs are shown in Figure 1. The proportion categorized as underweight, overweight, and obese were similar between the groups of mothers and fathers with around 5% being underweight, 30% being overweight and 5% being obese (Figure 1). In the additional categories with trigger points suggested for Asian populations, around 20% were at increased risk (BMI 23–24.9), and around 10% were at high risk (BMI 27.5–29.9). A table including this information can be found in the (Supplementary Table 1).

Results from the linear regression models can be found in Table 2. For the mothers, age was positively associated with BMI. Those mothers who were self-employed had a significantly higher BMI and those in the group of daily wage earners had a lower BMI compared to those without formal work. Women with previous pregnancies had a higher BMI compared to those being pregnant the first time. Those belonging to the Chhetri and the Tamang/Lama caste had significantly lower mean BMI compared to those belonging to the Newar caste. However, in the multivariable analyses, only age, and occupational group were retained as statistically significant. We could not find any association between maternal BMI and level of education, smoking status, family type, or number of family members. For the fathers included in this analysis, age, education level, and number of family members were positively associated with BMI. The BMI was higher in all occupational categories compared to daily wage earners, although it was not statistically significant for carpet workers. Fathers had a higher BMI if they lived in a joint family compared to a nuclear family. They had a lower BMI if they were in the Tamang/ Lama caste compared to the Newar caste. In the multivariate analysis, the results were similar to those for the mothers, where age and occupational group remained significantly associated with BMI. All other characteristics were not significantly associated with paternal BMI. Associations with BMI that were categorized as undernutrition or OWOB compared to a normal BMI showed a similar pattern (see Supplementary Tables 2, 3). These results should be interpreted cautiously due to the small sample size in some categories.

**Table 2:** Association between body mass index (BMI in kg/m^2^) of mothers and fathers and selected baseline characteristics, estimated in linear regression models.

**Characteristic**	**Mothers**			**Fathers**		
	***n***	**Coefficient (95% CI)**	**Adjusted coefficient**	***n***	**Coefficient (95% CI)**	**Adjusted coefficient**
			**(95% CI)**			**(95% CI)**
Age (years)	600	**0.25 (0.19, 0.31)**	**0.25 (0.19, 0.31)**	445	**0.16 (0.10, 0.23)**	**0.14 (0.08, 0.21)**
Education level	600	−0.01 (−0.21, 0.20)		445	**0.41 (0.13, 0.68)**	
**Occupation**						
Daily wage earner	75	Ref	Ref	166	Ref	Ref
No work/agriculture	373	**0.96 (0.07, 1.85)**	**0.99 (0.14, 1.83)**	28	**1.19 (−0.26, 2.64)**	0.77 (−0.66, 2.21)
Carpet worker	17	0.32 (−1.54, 2.20)	0.31 (−1.45, 2.08)	6	1.15 (−1.81, 4.11)	1.46 (−1.44, 4.37)
Self employed	73	**1.83 (0.69, 2.20)**	**1.67 (0.59, 2.75)**	137	**1.15 (0.33, 1.98)**	**0.95 (0.14, 1.76)**
Services	61	**1.74 (0.54, 2.95)**	**1.25 (0.10, 2.39)**	94	**1.71 (0.79, 2.63)**	**1.29 (0.37, 2.22)**
Working abroad	0	**–**	–	14	**3.38 (1.39, 5.36)**	**3.07 (1.12, 5.02)**
**Smoking**						
Yes	303	Ref		233	Ref	
No	297	0.30 (−0.27, 0.87)		212	−0.11 (−0.81, 0.57)	
**Alcohol consumption**						
Yes				322	Ref	
No				123	0.37 (−0.39, 1.14)	
**Gravida**						
Primi	281	Ref.				
>2	319	**1.41 (0.85, 1.98)**				
**Family type**						
Nuclear	308	Ref		222	Ref	
Joint	292	0.004 (−0.56, 0.57)		223	**0.49 (−0.19, 1.18)**	
Number of family members	600	0.07 (−0.05, 0.19)		445	**0.11 (−0.03, 0.25)**	
**Caste**						
Newar	422	Ref		336	Ref	
Brahmin	20	0.70 (−0.88, 2.29)		11	−1.00 (−3.23, 1.21)	
Chhetri	27	**−1.95 (−3.33**, **−0.57)**		17	**−1.55 (−3.35, 0.25)**	
Tamang/Lama	91	**−1.15 (−1.95,−0.34)**		54	**−0.77 (−1.83, 0.29)**	
Others	40	**−0.76 (−1.90, 0.38)**		27	0.48 (−0.96, 1.93)	

*The values in bold are significant at a level of p < 0.05*.

Anthropometric data were only available for 445 of 600 fathers. Those with missing BMI were slightly younger than those with information available (28.7 and 30.3 years; *p* = 0.05). They also had a lower education level with 46% being illiterate or with finished primary school compared to 31.7% in those with BMI available (*p* = 0.001).

## Discussion

In this study, we showed that in a group of young adults living in the municipality of Bhaktapur in Nepal, 5% were underweight (BMI <18.5) and 35% were overweight or obese (BMI ≥25). The anthropometric status was similar for men and women. There was a difference in mean BMI between different occupational groups and age was positively associated with BMI for both men and women.

Nepal is categorized as a low-income country according to the World Bank. However, in the last few decades, health indicators in Nepal have improved tremendously. For example, life expectancy at birth has increased from 54 years in 1990 to 70 years in 2017, and infant mortality rate (death per 1,000 live births) has decreased from 97.2 in 1990 to 26.7 in 2017 (30). At the same time, Nepal has still one of the highest prevalences of childhood stunting in the world. Nearly 40% of children under the age of five were reported with stunting in the Demographic Health Survey (DHS) 2016 (16). In addition to the very slow decline in stunting rates, there are reports of an increase in overweight and obesity (OWOB) (14, 31) and non-communicable diseases (NCDs) in Nepal (23, 24, 32). Comparing the prevalence of OWOB between the DHS published in 1995 and 2016, the prevalence was 4.2 times higher in 2016 with a higher increase noted for rural areas compared to urban (32). However, there remains a paucity of comprehensive and high-quality data on the double burden of malnutrition including both undernutrition and overweight in LMICs such as Nepal (33). Such information is critical for informing evidence-based health policies at the local and national levels.

In 2004, a WHO expert panel suggested additional trigger points to categorize BMI for Asian population based on higher observed risk for cardiovascular disease and diabetes mellitus type 2 compared to Caucasian populations at the same BMI (29). Possible reasons for an increased risk in Asian populations are a comparably higher percent of total body fat and a decreased fat-free mass at the same level of BMI (34). Asians have also been shown to be more prone to abdominal obesity and fat deposition in various organs such as the liver, which is strongly associated with insulin resistance (35, 36). At the same time, Zheng et al. (37) find that among 1 million Asian adults, those with a BMI between 22.6 and 27.5 had the lowest risk of mortality. In Indian and Bangladeshi cohorts, the risk of death was elevated for BMI <20 but not elevated at higher BMI levels. Despite these conflicting results, many Asian countries, such as India, have lowered their cut-off for defining obesity (38). In our study, 57% of the women and 54% for men would be defined as OWOB according to the cut-off of BMI ≥23 that is currently also used in the neighboring country India. This would not only mean an increase in the prevalence of OWOB, but also that more people at risk of obesity-related diseases. These would not be identified for treatment if only the WHO definition was used.

Our data is from adults measured between 2015 and 2017 who lived in the peri-urban municipality of Bhaktapur, which lies in the Kathmandu valley. Compared to other reports from Kathmandu and province 3 (including Kathmandu) (24, 39), the prevalence of both under- and overweight seems somewhat lower in our sample. This could be due to differences in mean age. We included relatively young adults with a mean age of around 30 years (compared to 40 years in the other reports). BMI usually increases with age, something we also showed in our analyses. The eligibility criteria of the original study might have also contributed to our findings, as only children with a height-for-age *Z*-score of <-1 were enrolled into the original study. However, there were few, only 41, children who did not comply with this inclusion criterion. Another consideration is that although women that are underweight have a higher risk of having offspring that is underweight, stunted, or micronutrient deficient, a study by Popkin et al. (11) showed that the global double burden of malnutrition within a household is primarily driven by the combination of overweight women with stunted children. Their result was also found for Nepal. Thus, we cannot infer a clear effect direction due to the exclusion of these 41 children and their parents.

In addition to age, occupational group was identified as being significantly associated with BMI in all our multivariate regression models. Occupation is closely related to other indicators of socio-economic status such as wealth, education, and caste and might not only indicate actual resources and thus access to health care and food, but also the level of physical activity/sedentary lifestyle. Daily wage earners, who on average had a lower BMI in our sample, usually have fewer resources and a more labor intense work compared to other occupational groups in Nepal. Education level was positively associated with BMI in the bivariable analyses, which agrees with other studies from Nepal (31, 39). However, we could only show an association between level of education and BMI in the fathers and not in the mothers. In a systematic review, Wei et al. (15) found that among women in Nepal, OWOB increased at all educational levels and that there was no clear pattern in underweight. Our findings could also indicate that in Nepal, men are often the main source of household income and therefore paternal education and occupation might contribute to a larger extent to the individual BMIs in the household compared to women's education level.

In our study sample, child undernutrition is still highly prevalent with 17% underweight and 32% stunted. Others report that 6.6% of 13–17 year old school children were overweight or obese in a national school survey (40) and 25.9% of 6–13 year old students in a private school in Lalitpur (41). Neither of these studies reported indicators of underweight. In the Lancet series on the double burden of malnutrition (DBM), Popkin et al. (11) describe a substantial increase in the prevalence of obese mothers with children that are stunted in Nepal. This emphasizes not only that malnutrition should be reported over the whole spectrum including both extremes of nutritional status, but also that intergenerational processes may play an important role (42), and thus, it underlines that we have an the opportunity and a need to explore critical windows for potential interventions so as to address several forms of malnutrition simultaneously.

### Strength and Limitations

For our analysis, we used data from parents whose children were enrolled into a community-based randomized-controlled trial. This will limit the generalizability of our results. In addition, mothers were in the post-pregnancy period (6–11 months after delivery), and potentially breastfeeding. Due to socio-cultural beliefs in Nepal, behavioral patterns during a child's first 6 months often change including ingesting a high calorie diet and having low physical activity levels (43). This could have distorted the BMI distribution. In this study, we did not have any data on diet and physical activity. Data were available for 445 fathers of the 600 children (74%) and thus we cannot exclude a selection bias. Those fathers with missing BMI were slightly younger and had a lower education level. This would have likely resulted in a slight overestimation of BMI as both age and educational level were positively associated with BMI in our sample. The data are cross-sectional, but participants were recruited over a period of 2 years, limiting the potential of seasonal effects on the nutritional status. Another important strength is that anthropometric measurements were taken by trained study staff and are not self-reported, thus represent high-quality data.

To conclude, our results contribute to documenting the whole distribution of BMI including both under- and overweight in a defined population of young adults living in a peri-urban community in Nepal. As Nepal undergoes an improvement in its economic situation and a nutrition transition, it is important to provide sufficient information based on data from different populations and potential target groups to enable timely health action and policies that are adapted to local needs and possibilities. In the 2020 Lancet series, Wells et al. (20) state that undernutrition and overweight show common drivers as well as physiological interactions and it is therefore important to identify these connections. Further research is needed to identify the main drivers of these conditions in other populations in Nepal or similar settings so as to provide a basis for developing potential interventions targeting malnutrition in all its forms simultaneously.

## Data Availability Statement

The raw data supporting the conclusions of this article will be made available by the authors, without undue reservation.

## Ethics Statement

The studies involving human participants were reviewed and approved by Nepal Health Research Council and Regional Committee for Medical and Health Research Ethics Norway. The patients/participants provided their written informed consent to participate in this study.

## Author Contributions

CS, RC, MU, and TS: conception/design of the work. RC, MU, and TS: data acquisition. CS: data analysis, data interpretation, and drafted the manuscript. RC, MU, MH, MS, TS, and SR: substantially revised the manuscript. All authors: approved the submitted version and agreed to be personally accountable for any part of the work.

## Conflict of Interest

The authors declare that the research was conducted in the absence of any commercial or financial relationships that could be construed as a potential conflict of interest.
