# Supplementary material for: Prevalence of Underweight, Overweight, and Obesity in Adults in Bhaktapur, Nepal in 2015–2017
Source: Front Nutr. 2020 Sep 22;7:567164. doi: 10.3389/fnut.2020.567164 (PMC7536337; doi:10.3389/fnut.2020.567164)
Supplement: Supplementary file 1 [file Data_Sheet_1.docx]

| **BMI** | **Mothers, n (%)** | **Fathers, n (%)** |
| --- | --- | --- |
| <18.5 | 34 (5.7) | 19 (4.3) |
| 18.5–22.9 | 223 (37.2) | 187 (42.0) |
| 23–24.9 | 134 (22.3) | 87 (19.5) |
| 25–27.4 | 122 (20.3) | 84 (18.9) |
| 27.5–29.9 | 53 (8.8) | 47 (10.6) |
| ≥ 30 | 34 (5.7) | 21 (4.7) |

**Supplementary material**

**Table S1**: Maternal and paternal body mass index (BMI; in m/kg^2^) categorized according to the commonly used cut-offs with additional trigger points suggested by WHO for Asian populations^1^

^1^ Consultation WHO. Appropriate body-mass index for Asian populations and its implications for policy and intervention strategies. Lancet. 2004;363(9403):157-63.

**Table S2**: Association of **mothers** body mass index ((BMI in m/kg^2^) and selected baseline characteristics, estimated in multinomial regression models with BMI categorized as underweight (BMI <18.5) and overweight/obese (BMI ≥ 25) compared to normal BMI (BMI 18.5-24.9).

| **Characteristic** | **Bivariable** | | | | **Multivariable** | |
| --- | --- | --- | --- | --- | --- | --- |
|  | **n** | **Underweight**  **RR (95% CI)** | **n** | **OWOB**  **RR (95% CI)** | **Underweight**  **RR (95% CI)** | **OWOB**  **RR (95% CI)** |
| Age (years) | 34 | **0.82 (0.74, 0.90)** | 209 | **1.11 (1.07, 1.15)** | **0.84 (0.76, 0.94)** | **1.11 (1.06, 1.15)** |
| Education level | 34 | 0.93 (0.71, 1.21) | 209 | 1.03 (0.91, 1.18) |  |  |
| Occupation  Daily wage earner  No work/ agriculture  Carpet worker  Self employed  Services | 6  23  1  2  2 | Ref  0.87 (0.33, 2.26)  0.75 (0.08, 6.94)  0.39 (0.07, 2.07)  0.49 (0.09, 2.57) | 17  131  5  31  25 | Ref  **1.75 (0.97, 3.17)**  1.33 (0.40, 4.40)  **2.17 (1.05, 4.45)**  **2.16 (1.01, 4.59)** | Ref  0.82 (0.29, 2.35)  0.69 (0.06, 6.96)  0.43 (0.07, 2.52)  0.88 (0.15, 5.21) | Ref  **1.94 (1.05, 3.59)**  1.43 (0.39, 5.17)  **2.23 (1.05, 4.72)**  1.90 (0.87, 4.17) |
| Smoking  Yes  No | 18  16 | Ref  0.95 (0.47, 1.93) | 100  109 | Ref  1.17 (0.83, 1.64) |  |  |
| Gravida  Primi  ≥2 | 24  10 | Ref  **0.42 (0.19, 0.91)** | 77  132 | Ref  1.74 (1.22, 2.47) |  |  |
| Family type  Nuclear  Joint | 20  14 | Ref  0.74 (0.36, 1.52) | 104  105 | Ref  1.07 (0.76, 1.51) |  |  |
| Number of family members | 34 | **0.82 (0.67, 1.00)** | 209 | 1.02 (0.95, 1.10) |  |  |
| Caste  Newar  Brahmin  Chhetri  Tamang/ Lama  Others | 13  3  7  9  2 | Ref  **6.33 (1.52, 26.2)**  **8.86 (3.08, 25.4)**  **3 (1.22, 7.35)**  1.31 (0.28, 6.09) | 162  8  5  25  9 | Ref  1.35 (0.51, 3.58)  0.50 (0.18, 1.42)  **0.66 (0.40, 1.11)**  **0.47 (0.21, 1.02)** | Ref  **5.72 (1.34, 24.2)**  **5.04 (1.60, 15.8)**  1.64 (0.57, 4.70)  0.77 (0.15, 3.84) | Ref  1.55 (0.57, 4.21)  0.72 (0.25, 2.10)  1.05 (0.58, 1.89)  0.66 (0.29, 1.50) |

**Table S3:** Association of **fathers** body mass index ((BMI in m/kg^2^) and selected baseline characteristics, estimated in multinomial regression models with BMI categorized as underweight (BMI <18.5) and overweight/obese (BMI ≥ 25) compared to normal BMI (BMI 18.5-24.9).

| **Characteristic** | **Bivariable** | | | | **Multivariable** | |
| --- | --- | --- | --- | --- | --- | --- |
|  | **n** | **Underweight**  **RR (95% CI)** | **n** | **OWOB**  **RR (95% CI)** | **Underweight**  **RR (95% CI)** | **OWOB**  **RR (95% CI)** |
| Age (years) | 19 | **0.93 (0.83, 1.03)** | 152 | **1.07 (1.03, 1.11)** | **0.92 (0.82, 1.03)** | **1.06 (1.02, 1.11)** |
| Education level | 19 | 0.83 (0.55, 1.24) | 152 | **1.18 (1.01, 1.39)** |  |  |
| Occupation  Daily wage earner  No work/ agriculture  Carpet worker  Self employed  Services  Working abroad | 9  0  0  5  5  - | Ref  -  -  0.79 (0.25, 2.47)  1.27 (0.40, 4.00)  - | 42  9  2  52  39  8 | Ref  1.29 (0.54, 3.08)  1.36 (0.24, 7.75)  **1.77 (1.08, 2.92)**  **2.13 (1.23, 3.69)**  **3.65 (1.19, 11.1)** | Ref  -  -  0.88 (0.28, 2.78)  1.60 (0.48, 5.30)  - | Ref  1.08 (0.44, 2.63)  1.59 (0.27, 9.26)  1.65 (0.99, 2.73)  **1.81 (1.03, 3.17)**  **3.34 (1.07, 10.4)** |
| Smoking  Yes  No | 11  8 | Ref  0.81 (0.31, 2.09) | 91  61 | Ref  **0.75 (0.50, 1.12)** |  |  |
| Alcohol consumption  Yes  No | 12  7 | Ref  1.57 (0.59, 4.15) | 110  42 | Ref  1.03 (0.66, 1.60) |  |  |
| Family type  Nuclear  Joint | 8  11 | Ref  1.59 (0.62, 4.07) | 67  85 | Ref  **1.46 (0.98, 2.18)** |  |  |
| Number of family members | 19 | **1.14 (0.95, 1.36)** | 152 | **1.06 (0.98, 1.15)** |  |  |
| Caste  Newar  Brahmin  Chhetri  Tamang/ Lama  Others | 13  1  1  3  1 | Ref  2.21 (0.25, 19.4)  1.19 (0.14, 9.85)  1.29 (0.35, 4.77)  0.97 (0.11, 7.90) | 121  3  3  15  10 | Ref  0.71 (0.18, 2.81)  **0.38 (0.10, 1.37)**  0.69 (0.36, 1.32)  1.04 (0.45, 2.37) |  |  |
